# Uncovering essential anesthetics-induced exosomal miRNAs related to hepatocellular carcinoma progression: a bioinformatic investigation

**DOI:** 10.1186/s12920-024-01922-7

**Published:** 2024-06-05

**Authors:** Ning Huang, Jie Fang, Fang Du, Jichuan Zhou, Yuxin Li, Xiaoguang Zhang

**Affiliations:** Department of anesthesia, Zhongshan Hospital, Fudan University, 180 Fenglin Road, Shanghai, China

**Keywords:** Hepatocellular carcinoma, Anesthetic, Sevoflurane, Exosome, miRNA, miR-21-4-5p, Tumor microenvironment

## Abstract

**Background:**

Anesthetic drugs may alter exosomal microRNA (miRNA) contents and mediate cancer progression and tumor microenvironment remodeling. Our study aims to explore how the anesthetics (sevoflurane and propofol) impact the miRNA makeup within exosomes in hepatocellular carcinoma (HCC), alongside the interconnected signaling pathways linked to the tumor immune microenvironment.

**Methods:**

In this prospective study, we collected plasma exosomes from two groups of HCC patients (*n* = 5 each) treated with either propofol or sevoflurane, both before anesthesia and after hepatectomy. Exosomal miRNA profiles were assessed using next-generation sequencing (NGS). Furthermore, the expression data from The Cancer Genome Atlas-Liver Hepatocellular Carcinoma (TCGA-LIHC) was used to pinpoint the differentially expressed exosomal miRNAs (DEmiRNAs) attributed to the influence of propofol or sevoflurane in the context of HCC. Gene set enrichment analysis (GSEA) and gene set variation analysis (GSVA) were used to dissect the signaling pathways and biological activities associated with the identified DEmiRNAs and their corresponding target genes.

**Results:**

A total of 35 distinct DEmiRNAs were exclusively regulated by either propofol (*n* = 9) or sevoflurane (*n* = 26). Through TCGA-LIHC database analysis, 8 DEmiRNAs were associated with HCC. These included propofol-triggered miR-452-5p and let-7c-5p, as well as sevoflurane-induced miR-24-1-5p, miR-122-5p, miR-200a-3p, miR-4686, miR-214-3p, and miR-511-5p. Analyses revealed that among these 8 DEmiRNAs, the upregulation of miR-24-1-5p consistently demonstrated a significant association with lower histological grades (*p* < 0.0001), early-stage tumors (*p* < 0.05) and higher survival (*p* = 0.029). Further analyses using GSEA and GSVA indicated that miR-24-1-5p, along with its target genes, were involved in governing the tumor immune microenvironment and potentially inhibiting tumor progression in HCC.

**Conclusions:**

This study provided bioinformatics evidence suggesting that sevoflurane-induced plasma exosomal miRNAs may have a potential impact on the immune microenvironment of HCC. These findings established a foundation for future research into mechanistic outcomes in cancer patients.

**Supplementary Information:**

The online version contains supplementary material available at 10.1186/s12920-024-01922-7.

## Background

Liver cancer ranked as the third primary contributor to cancer-related fatalities in 2020, with hepatocellular carcinoma (HCC) being the predominant form of primary liver cancer across the world [1]. Surgical resection has been the mainstay curative treatment for HCC, and other treatment modalities including liver transplantation and radiofrequency ablation [2]. Despite the progress made in early detection and the implementation of (neo)adjuvant treatments, a significant number of HCC patients remain susceptible to the recurrence and spread of tumors [3]. These circumstances underscore the imperative need to comprehend the underlying mechanisms driving HCC tumor progression.

In recent years, the impact of anesthetic drugs on tumor progression has received increasing attention from researchers [4]. Clinical investigations have indicated that the selection of anesthesia can potentially modify both immune functionality and the progression of tumors [5–8]. For instance, retrospective clinical studies carried out on HCC patients reported that patients administered with propofol (intravenous anesthetic) exhibited decreased cancer recurrence rates and enhanced survival outcomes in contrast to those who received volatile anesthetics [7]. In addition, Yan et al. found that sevoflurane, an inhalation anesthetic, increased circular vascular endothelial growth factor (VEGF) after breast cancer surgery [9], which was believed to promote the growth and resurgence of tumors [10]. Contrarily, several in vitro studies have suggested that sevoflurane might possess an anti-tumor impact on HCC [11–13]. These conflicting viewpoints might stem from factors such as small sample sizes, the diversity of tumors, and inherent retrospective biases. In recent years, there has been an increasing number of prospective clinical studies examining the long-term effects of various anesthetic drugs on tumor prognosis. Nevertheless, a consensus on the impact of anesthetics on the long-term prognosis of cancer patients has yet to be reached. A long-term follow-up study involving 1764 breast cancer patients revealed no significant difference in the 5-year survival rate between those administered with propofol and sevoflurane anesthesia [14]. In 2023, the researchers of the First Study of Perioperative Organ Protection (SPOP1) discovered that follow-up data for elderly cancer patients after surgery indicated that the use of propofol or sevoflurane anesthesia did not affect the overall survival rates [15]. However, a study focusing on patients with breast intraductal carcinoma undergoing breast-conserving surgery indicated that individuals under propofol-based anesthesia had a lower risk of local recurrence compared to those who received sevoflurane anesthesia [16]. Consequently, the influence of anesthetic drugs on cancer prognosis still requires further clarification.

Emerging evidence acknowledges exosomes as critical regulators in cancer initiation, progression, metastasis, and immune evasion through the remodeling of tumor microenvironment [17]. Exosomes, ranging from 30 to 150 nm, are extracellular carriers that envelop enzymes, ribosomes, cytokines, and diverse RNA types, encompassing messenger RNA (mRNA), long non-coding RNA (lncRNA), and microRNA (miRNA). The contents within exosomes facilitate intercellular communication and influence the microenvironment of cells by modifying signaling pathways within recipient cells. Particularly, miRNAs have been identified as playing roles either as tumor suppressors or oncogenes in the progression of tumors [18].

Numerous research efforts have attempted to unravel the implications of anesthetics on exosomal functions. For instance, Wang et al. exhibited that propofol can enhance HCC advancement by influencing exosomal lncRNA H199 [19]. He et al. showed that exosome-mediated circular RNA (Circ‑HMGCS1) suppresses cell viability and invasion in colon cancer treated with sevoflurane [20]. Another study by Buschmann et al. demonstrated that propofol and sevoflurane reduced the concentration of circulating vesicles and observed changes in miRNA contents during colorectal cancer resection [21]. Nevertheless, the precise manner in which distinct anesthetic drugs may modify the miRNA components within exosomes in the context of HCC remains unknown.

Therefore, our current study aims to investigate the effects of different anesthetics on exosome miRNA profiles in HCC patients. We delved into the correlation between these exosomal miRNAs and the prognostic outcomes among HCC patients. Additionally, we investigated the plausible target genes influenced by anesthetic-triggered miRNAs and examined the roles of miRNAs and their corresponding targets in the progression of HCC. Our study offers initial insight into the potential effects of exosomes influenced by anesthetics on the proliferation and metastasis of HCC.

## Methods

### Ethics and patient enrollment

This study was approved by the Ethics Committee of Zhongshan Hospital affiliated with Fudan University. All participants signed written informed consent forms prior to enrollment. The study was carried out according to the World Medical Association Declaration of Helsinki. All samples utilized in the study were subjected to anonymization protocols during the analysis process. Between October 2020 and July 2021, participants were recruited from among those patients scheduled to undergo laparoscopic liver resection surgery at Zhongshan Hospital affiliated with Fudan University. The night before the surgery, a standard preoperative assessment was conducted by the designated anesthesiologist. Written informed consent for study participation and anesthesia were obtained separately.

The inclusion criteria were (1) male patients with primary HCC scheduled for laparoscopic hepatectomy; (2) age 30–55 years; (3) American Society of Anesthesiologists (ASA) physical status ≤ 2; (4) single tumor, size ≤ 5 cm, no tumor thrombus or distant metastasis of abdominal lymph nodes; (5) Child-Pugh class A and Desmet-Scheuer score < F3; and (6) No alternative treatments (e.g. chemotherapy, interventional therapy, etc.) were administered for liver cancer.

Exclusion criteria were (1) lack of consent; (2) individuals with taboos that restricted the usage of intravenous or inhalation anesthetics; (3) laparoscopic procedures that were converted to open surgery; (4) patients who needed immediate intervention due to their unstable vital signs resulting from significant intraoperative bleeding; (5) pathological examination revealed that the size, kind, or stage of the tumor was not eligible for inclusion; and (6) participant withdrawal.

The study was structured as a prospective, non-randomized preliminary inquiry. The patient recruitment procedure is visually depicted in Supplementary Figure S1. Ultimately, the study cohort comprised five patients anesthetized using propofol and an additional five individuals anesthetized with sevoflurane.

### Anesthetic method and sample collection

All patients received a standard balanced anesthetic consisting of either opioid plus propofol or sevoflurane. During the operation, sufentanil was used as the primary analgesic, and rocuronium was used as the muscle relaxant. The induction dose of rocuronium is 0.6 mg/kg. Sufentanil and rocuronium were added as needed during the operation. At the end of the surgery, residual muscle relaxation was antagonized with sugammadex. Propofol group was induced and maintained with target-controlled infusion of propofol and remifentanil. The sevoflurane group underwent induction with a combination of sevoflurane inhalation and 1 µg/kg remifentanil. Alternatively, maintenance was carried out solely with sevoflurane. The decision regarding the anesthetic agent was at the discretion of the attending anesthesiologist, adhering to the established protocols at our institution.

Venous blood was drawn through central venous catheters 30 min before anesthesia and 24 h after termination of surgery. Ten milliliter serum tubes (BD Vacutainer™ EDTA Blood Collection Tubes) were used for blood collection. Samples stood at 4 °C for 3 ∼ 4 h and centrifuged at 3,400×g for 10 min at 4 °C. Serum was aliquoted and stored at − 80 °C until further processing.

### Exosome isolation, RNA extraction, library preparation, small RNA sequencing and data analysis

Exosomes were isolated from plasma using the RiboTM Exosome Isolation Reagent (Ribobio, China), and exosomal RNAs were extracted using MagZol reagent (Magen) according to the manufacturer’s protocol. The quantity and integrity of exosomal RNA yield were assessed using the Qubit®2.0 (Invitrogen, USA) and Agilent 2200 TapeStation (Agilent Technologies, USA) separately. The RNAs were ligated with 3’ RNA adapter, followed by 5’ adapter. The adapter-ligated RNAs were subjected to RT-PCR and amplified at low cycle numbers. Then, the PCR products were subjected to size selection using polyacrylamide gel electrophoresis (PAGE), according to the guidelines outlined in the NEBNext® Multiplex Small RNA Library Prep Set for Illumina® (Illumina, USA). The fragment size of the purified library products underwent assessment using the Agilent 2200 TapeStation. The libraries were sequenced in a single-end manner with a read length of 50 base pairs (SE50) on the HiSeq 2500 platform (Illumina, USA) at Ribobio Co. Ltd (Ribobio, China).

Clean reads were obtained by filtering out raw reads containing adapter, poly ’N’, of low quality, and were smaller than 17nt reads using FASTQC. Clean reads were sequentially aligned to reference sequences of human noncoding RNAs using BWA. miRDeep2 was used to identify known mature miRNA based on miRBase21 and to predict the novel miRNAs. Databases of Rfam12.1 and pirnabank were used to identify rRNA, tRNA, snRNA, snoRNA, and piRNA by BLAST. The miRNA expressions were calculated and normalized to reads per million (RPM) values [RPM=(number of reads mapping to miRNA/ number of reads in clean data)×10^6^ ]. Differentially expressed miRNAs (DEmiRNAs) between the paired pre- and post-operation sample sets were computed using the edgeR algorithm, according to the criteria of |log_2_Fold Change (FC)| > 1 and adjusted *p*-value < 0.05. Only the anesthetic-induced DEmiRNAs that fulfilled the stringent filtering criteria (RPM ≥ 50 reads; |log_2_(FC)|>1 and adjusted *p*-value < 0.01) were eligible for subsequent analyses.

### Bioinformatic analysis of DEmiRNAs in primary HCC

#### Data source of primary HCC and pre-processing

Transcriptome profiles and clinical information on HCC were downloaded from The Cancer Genome Atlas-Liver Hepatocellular Carcinoma (TCGA-LIHC) which contained 374 tumor and 50 normal samples. Based on the “limma” R package with selection criteria of |log_2_FC|>0.5 and adjusted *p-*value < 0.05, the differential expressions of miRNAs and mRNAs in TCGA-LIHC were identified. Venn diagrams were used to visualize the intersections between the DEmiRNAs and miRNAs from the TCGA dataset. The volcano plot and the corresponding heatmap were generated using the “pheatmap” and “ggplot2” R packages. Boxplots and correlation plots were generated in R using package “ggpubr”. Survival analysis was performed using “survival” and “survminer” R packages. TargetScan and miRDB were used to predict the target genes of selected miRNA. The associations between miRNAs and target genes were illustrated using CytoScape.

#### Functional and pathway enrichment analyses

Pathway enrichment analysis was conducted using the gene set enrichment analysis (GSEA) algorithms and gene set variation analysis (GSVA). GSEA was performed using the “clusterProfiler” package in R. Corresponding pathway databases include Gene Ontology (GO), Kyoto Encyclopedia of Genes and Genomes (KEGG), and Reactome. GSVA analysis was performed using the GSVA package in R, and the relevant biological activity was assessed based on the Hallmark gene sets of MsigDB database, which included 50 biological processes closely associated with tumors. GSVA scoring was first performed on all tumor samples in TCGA-LIHC, and the correlation between the specific gene expression and the pathway score in HCC was calculated.

Several gene sets curated by Mariathasan and colleagues [22] were used to examine the correlation between DEmiRNAs and biological processes associated with the tumor microenvironment, including (i) CD8^+^ T-effector signature [23]; (ii) antigen processing machinery [24]; (iii) immune-checkpoint; (iv) epithelial-mesenchymal transition (EMT) markers previously reported [25]; (v) pan-fibroblast TGF-β response signature (Pan-F-TBRS); (vi) DNA replication-dependent histones [22]; (vii) select members of the DNA damage repair-relevant gene set [26]; (viii) mismatch repair (KEGG); (ix) nucleotide excision repair (KEGG); and (x) base excision repair.

The genetic and epigenetic alterations of the DEmiRNA targets, specifically the copy number variation (CNV) and methylation, were conducted using the Gene Set Cancer Analysis (GSCA) database. The association between these genes and HCC immune infiltration was also analyzed with the GSCA database [27].

### Statistics

Variables were tested for normal distribution using the Shapiro-Wilk normality test. For comparison between two groups, normally distributed variables were tested using an unpaired Student’s t-test, while non-normally distributed variables were tested using the Wilcoxon rank sum test. Differences in categorical values between groups were compared using Fisher´s exact test. A value of *p* < 0.05 was regarded as statistically significant. All statistical analysis was performed using SPSS Version 26.0 (SPSS, USA) and R statistical software, version 4.1.1.

## Results

### Patient characteristics and variables

We recruited 10 male patients, with 5 patients receiving propofol and 5 receiving sevoflurane. The propofol group had a median age of 50 (IQR: 43.5–51.5), while those in the sevoflurane group had a median age of 44 (IQR: 36-54.5). There was no significant difference in age between the two groups (*p* = 0.594). Only male patients were chosen because male predominates among HCC patients [28], and also to mitigate the potential influence of gender variations on the sequencing outcomes. Similarly, no significant differences were observed between the two groups in terms of body mass index, ASA classification ratio, histologic inflammation grade, fibrosis stage, and tumor stage (*p* > 0.05) (Table 1). Furthermore, other variables such as opioid use, blood loss, and the duration of surgery between the two groups, showed no significant differences between the two groups (*p* > 0.05) (Table 2).

**Table 1:** **Correlation analysis between clinical characteristics and anesthetics**

Parameter	propofol	sevoflurane	*p*-value
Age (yr)*	50(43.5–51.5)	44(36-54.5)	0.594
Body mass index (kg/m^2^)*	20.8(19.4–24.2)	21.3(19.4–26.6)	0.642
ASA Physical Status (I/II)	2/3	2/3	1.000
Tumor size (cm^3^)*^#^	9.8(2.1–49.3)	16.4(6.6–31.7)	0.490
Tumor stage (I/II/III)†	0/4/1	0/4/1	1.000
BCLC stage (0/A/B/C/D)^§^	0/5/0/0/0	0/5/0/0/0	1.000
Inflammatory grade by Scheuer scoring system (1/2)	1/4	2/3	0.513
Fibrotic stage by Scheuer scoring system (1/2/3/4)	0/0/2/3	1/0/2/2	0.419
HBV infection (yes/no)	5/0	5/0	1.000
Anti-viral treatments to HBV (yes/no)	3/2	2/3	0.500
Liver stiffness measurement by liver B-ultrasound (kPa)*	12.8(11.0-14.2)	11.8(7.5–16.4)	0.756
Preoperative platelet count (×10^9^/L)*	150(124–164)	204(118–247)	0.253
Preoperative AFP level (ng/mL)*	667(180–3147)	32(4-415)	0.117

*Data are median (p25 – p75). ASA, American Society of Anesthesiologists; HBV, hepatitis B virus; AFP, alpha-fetoprotein. ^#^Tumor size (cm^3^)=π/6×length(cm)×width(cm)×heigth(cm). †The Edmondson-Steiner grading system for HCC. §The Barcelona Clinic Liver Cancer (BCLC) staging system

**Table 2:** Comparison of intraoperative drug and surgical conditions between the propofol and sevoflurane group

Parameter	propofol	sevoflurane	*p*-value
Sufentanil (ug)	35(27.5–40)	40(37.5–60)	0.077
Blood loss (ml)	50(50–125)	100(750 − 150)	0.174
Duration of surgery (min)	90(75–141)	135(85–178)	0.384

Data are median (p25 – p75)

### Detection of differentially expressed exosomal miRNAs (DEmiRNAs) in response to anesthetics

To identify the exosomal miRNAs with altered expression in response to propofol or sevoflurane administration, we performed DEmiRNA expression analysis of paired samples in either group. Employing the pre-operative data as the control, we identified 237 DEmiRNAs via edgeR algorithm based on |log_2_FC|>1 and adjusted *p-*value < 0.05. Within these, 94 DEmiRNAs were identified in the propofol group, while 143 were observed in the sevoflurane group (Supplemental Figure S2).

Using stringent filtering criteria for miRNA expression level (RPM ≥ 50 reads, |log_2_FC|>1, and adjusted *p*-value < 0.01) and by filtering out the miRNAs with opposite trends between individuals, 22 DEmiRNAs were found to be significantly regulated by propofol (14 up, log_2_FC range = 1.68 to 4.48; 8 down, log_2_FC range = − 1.77 to − 5.91) and 39 DEmiRNAs by sevoflurane (30 up, log_2_FC range = 1.26 to 5.52; 9 down, log_2_FC range = − 1.25 to − 2.85) (Supplemental Table S1).

**Fig. 1:**
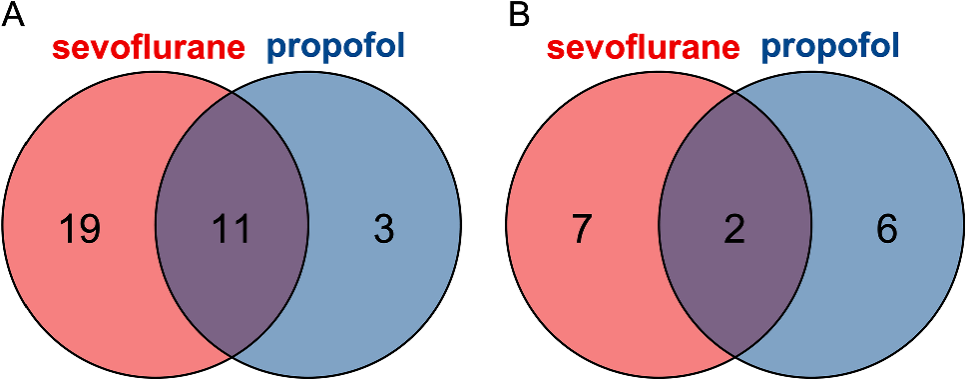
Differential regulation of DEmiRNAs by propofol and sevoflurane. (**A**) Venn diagram of DEmiRNAs significantly upregulated by different anesthetic drugs after surgery. (**B**) Venn diagram of DEmiRNAs significantly downregulated by different anesthetic drugs after surgery

Next, we assessed potentially overlapping DEmiRNA expression levels between the propofol and sevoflurane groups. As demonstrated in Fig. 1, both groups shared 11 upregulated DEmiRNAs and 2 downregulated DEmiRNAs. These overlapping DEmiRNAs were excluded from the following analysis because the alterations in these miRNAs could be the consequences of surgery instead of anesthetics. A total of 35 DEmiRNAs were exclusively regulated: 3 were specifically upregulated and 6 were downregulated by propofol, while 19 were upregulated and 7 were downregulated by sevoflurane.

### Differential expression analysis of DEmiRNAs in TCGA-LIHC

**Fig. 2:**
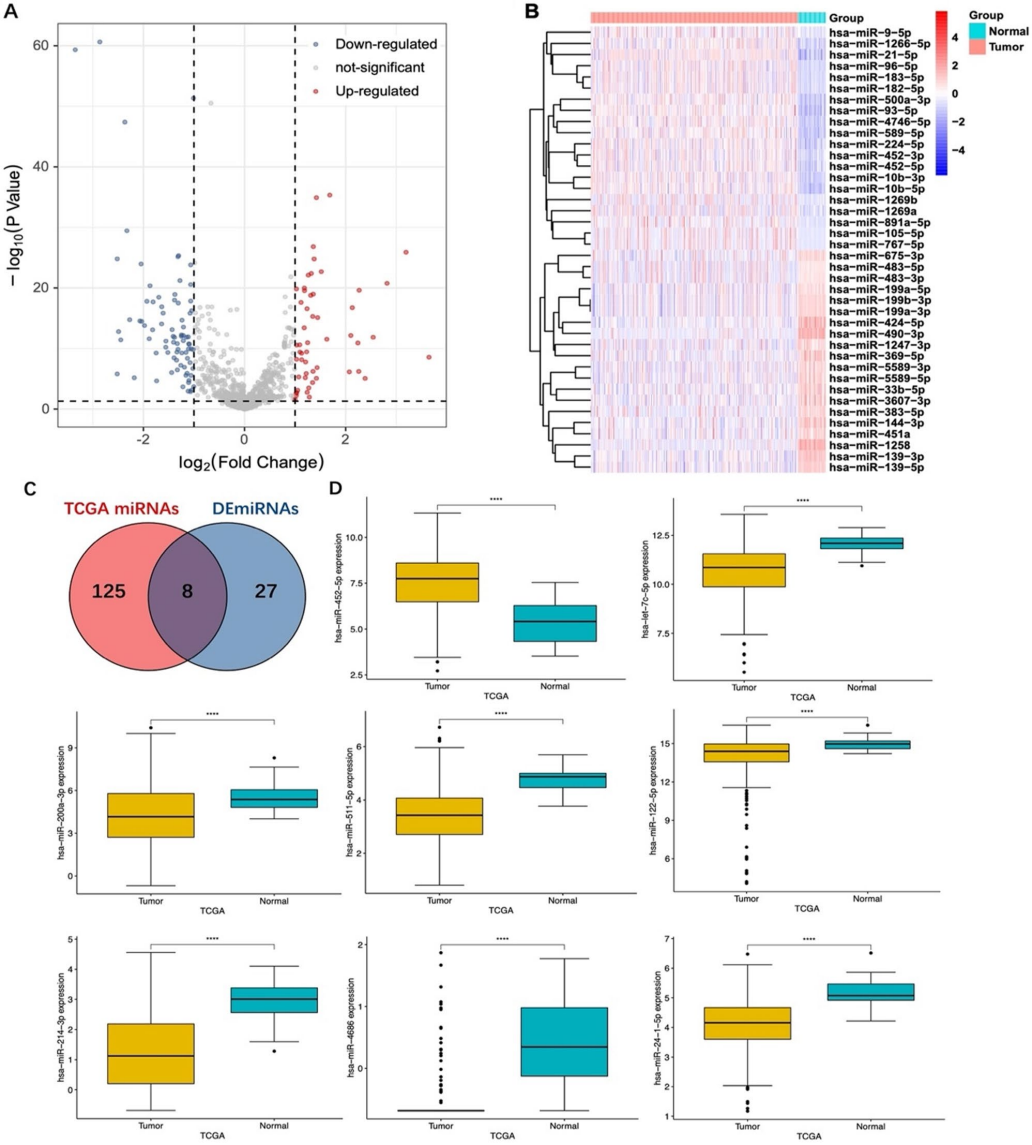
Different expressions of 8 DEmiRNAs in TCGA-LIHC. (**A**) Volcano plot of differentially expressed miRNAs in the TCGA-LIHC (TCGA-miRNAs) cohort. The red dot represented the upregulated miRNA, while the blue dot represented the downregulated miRNA. (**B**) Heatmap of the differentially expressed miRNAs in 374 LIHC and 50 normal tissues from the TCGA database. The color bar from red to blue denotes high to low gene expression. (**C**) Venn diagram of 8 DEmiRNAs overlapped with TCGA-LIHC differentially expressed miRNAs. (**D**) The expression patterns of 8 DEmiRNAs between tumor and normal tissues in TCGA-LIHC cohort. ****p < 0.0001

To investigate the role of DEmiRNAs in HCC specifically induced by anesthetic, we analyzed the DEmiRNAs using the TCGA-LIHC database. We identified 133 differentially expressed miRNAs (TCGA miRNAs) between tumor and normal tissues using the “limma” package with the screening criteria of |log_2_FC|>0.5 and adjusted *p* < 0.05 (Fig. 2A, B). Eight TCGA miRNAs overlapped with the DEmiRNAs specifically regulated by propofol or sevoflurane (Fig. 2C).

Of the 8 overlapping DEmiRNAs, 2 belonged to the propofol group (upregulation of miR-452-5p, and downregulation of let-7c-5p) and 6 belonged to the sevoflurane group (upregulation of miR-24-1-5p, miR-122-5p, miR-200a-3p, miR-4686, miR-214-3p, miR-511-5p) (Supplemental Table S1). Analysis of these DEmiRNAs expression patterns in the TCGA-LIHC cohort showed that miR-452-5p expression was significantly increased in HCC compared to the normal tissue (*p* < 0.0001), while the other 7 DEmiRNAs were significantly suppressed in HCC (*p* < 0.0001) (Fig. 2D). Taken together, our results possibly suggest that propofol-triggered miR-452-5p and let-7c-5p may be associated with HCC progression, while sevoflurane-induced miR-24-1-5p, miR-122-5p, miR-200a-3p, miR-4686, miR-214-3p, miR-511-5p may be associated with HCC suppression.

### Correlation between the DEmiRNAs and HCC prognosis

**Fig. 3:**
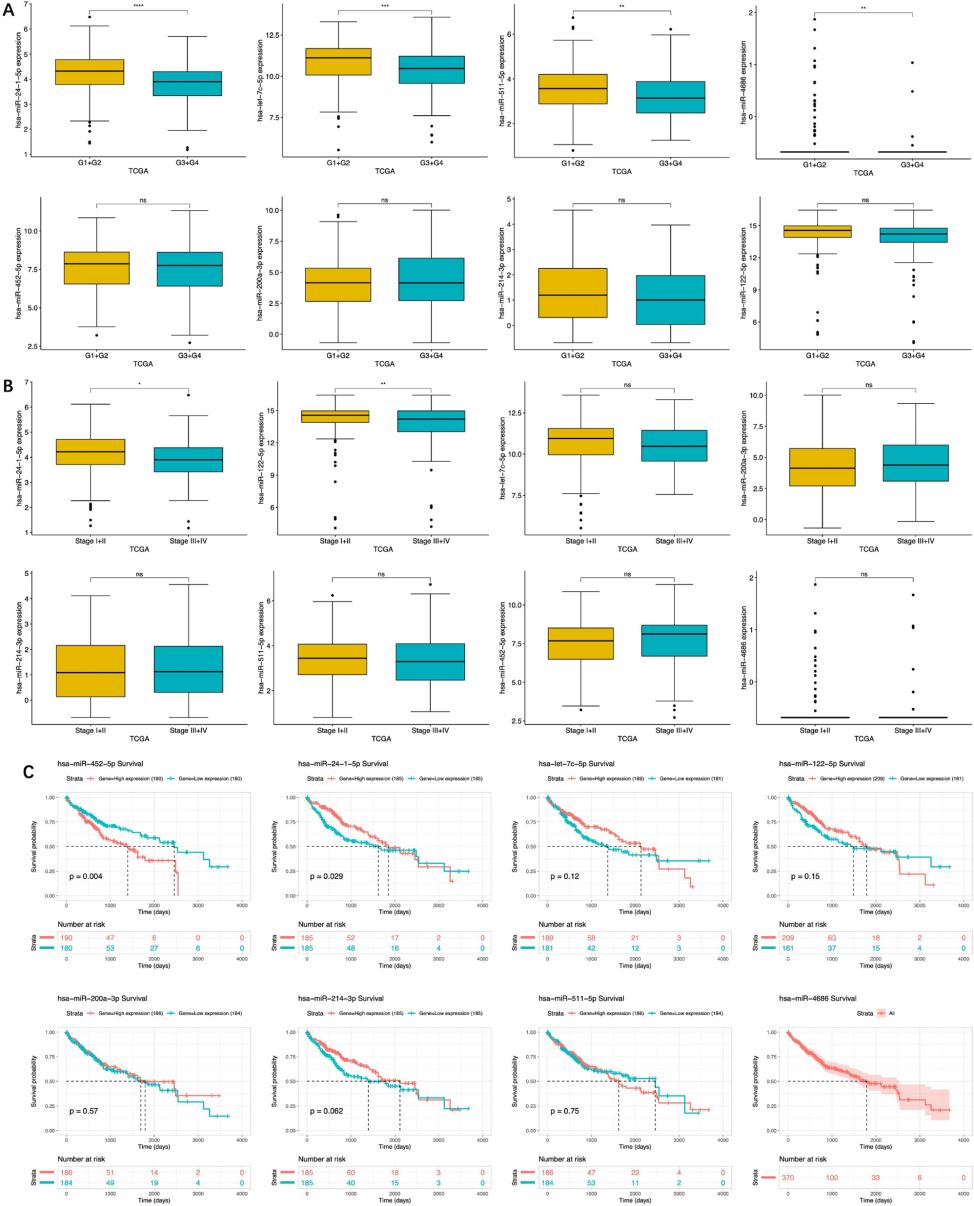
Correlation between DEmiRNA expression levels and HCC grade, stage and median survival time. (**A**) Expression of DEmiRNA in different histological grades of HCC. (**B**) Expression of DEmiRNA in different pathological stages of HCC. (**C**) Correlation of the DEmiRNA with median survival of HCC patients. *p < 0.05; **p < 0.01; ***p < 0.001; ****p < 0.0001

To assess the impact of the eight DEmiRNAs on the occurrence and progression of HCC, the expression of these miRNAs were analyzed based on the histological grades and tumor stages of TCGA-LIHC datasets. Of the 8 DEmiRNAs, only the upregulation of miR-24-1-5p was significantly associated with both low histological grades (*p* < 0.0001) and early-stage tumors (*p* < 0.05) (Fig. 3A, B). Upregulation of sevoflurane-induced miR-214-3p and miR-200a-3p showed no significant effect in both clinical parameters, and the other DEmiRNAs upregulated by sevoflurane were only significantly associated with either low histological grades (miR-511-5p (*p* < 0.01), and miR-4686 (*p* < 0.01)) or early-stage tumors (miR-122-5p (*p* < 0.01)). The upregulated propofol-induced miR-452-5p showed no significant effect on both the histological grades and tumor stages, while the downregulated let-7c-5p was only significantly associated with low histological grades (*p* < 0.001) (Fig. 3A, B).

For further analysis, the TCGA-LIHC data were divided into high and low expressions, and survival analysis was performed according to the overall expression level of the DEmiRNAs. Results showed that HCC patients with high expression of miR-24-1-5p had a significantly higher survival compared to those with low expression (*p* = 0.029), and those with high expression of miR-452-5p had a significantly lower survival (*p* = 0.004) (Fig. 3C). Taken together, only the expressions of sevoflurane-induced miR-24-1-5p were consistent, and the upregulation of miR-24-1-5p was associated with better prognosis in HCC patients.

### Predicted target genes of mir-24-1-5p in HCC

**Fig. 4:**
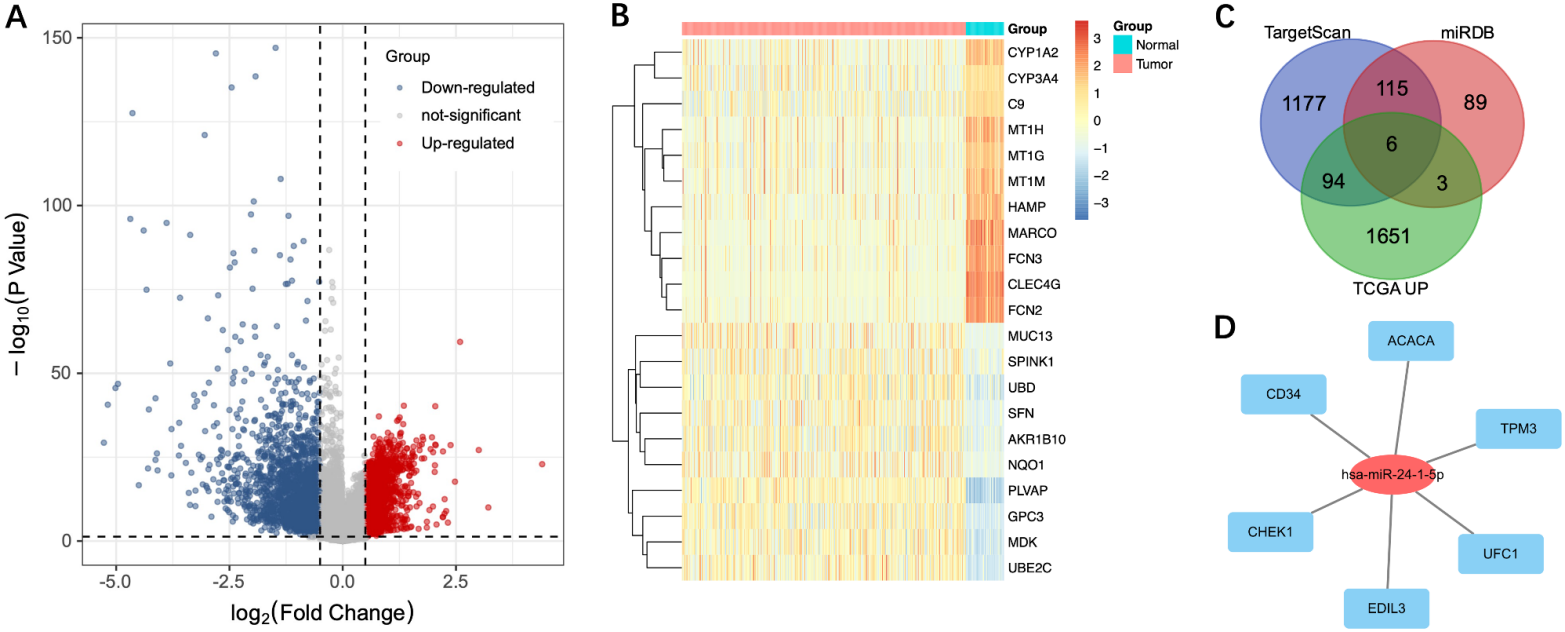
Prediction of miR-24-1-5p targets. (**A**) Volcano map of differentially expressed genes between tumor and normal tissues in TCGA-LIHC. (**B**) Heatmap of differentially expressed genes between tumor and normal tissues in TCGA-LIHC. (**C**) Venn diagram of predicted target genes of miR-24-1-5p overlapped with TCGA-LIHC upregulated mRNAs. (**D**) The interaction network of miR-24-1-5p and its key target genes in HCC

As the high expression of miR-24-1-5p is associated with a better prognosis, we next sought to determine its specific mRNA targets in HCC. Using log_2_FC > 0.5 and adjusted *p* < 0.05 as the screening criteria, we performed a differential analysis of gene expression in TCGA-LIHC samples and identified 1754 genes that were significantly upregulated in HCC samples (Fig. 4A, B). We then predicted the miR-24-1-5p targets using TargetScan and miRDB database and obtained 1392 and 213 candidates, respectively. To improve the reliability of the predicted targets, we intersected the predicted targets with the upregulated genes expressed in HCC samples and obtained 6 overlapping candidates (CD34, ACACA, TPM3, UFC1, EDIL3, and CHEK1) (Fig. 4C). Using Cytoscape software, the network of miR-24-1-5p targets is presented in Fig. 4D.

### Signaling pathway correlated with mir-24-1-5p in HCC

**Fig. 5:**
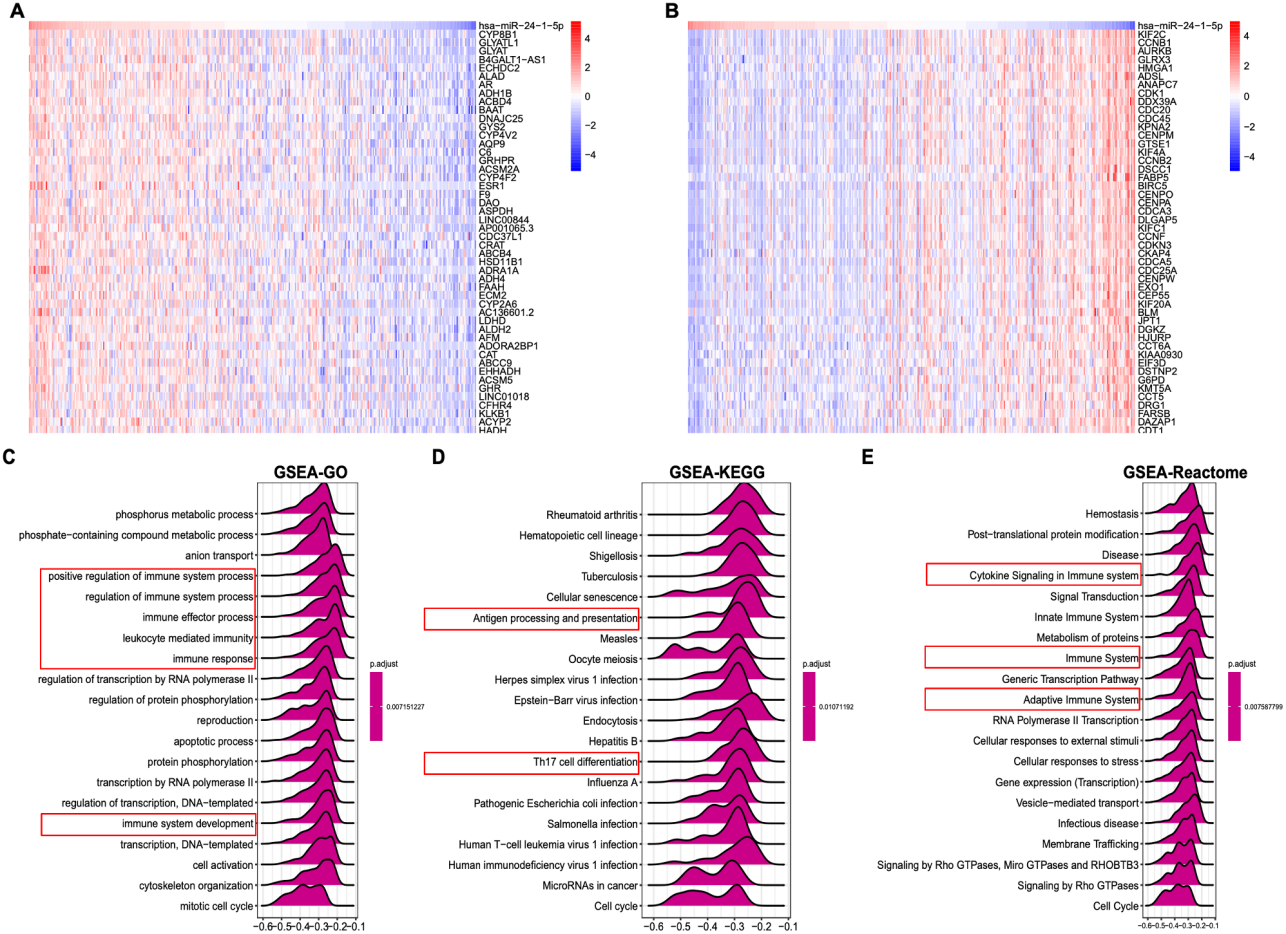
Enrichment analysis of genes correlated with miR-24-1-5p in HCC. (**A**) Heatmap of top 50 genes with significant positive correlations with miR-24-1-5p in TAGC-LIHC data. (**B**) Heatmap of top 50 genes with significant negative correlations with miR-24-1-5p in TAGC-LIHC data. (**C**) GO analysis. (**D**) KEGG analysis. (**E**) Reactome analysis

To analyze the biological functions and pathways affected by miR-24-1-5p, we performed a correlation analysis between miR-24-1-5p and gene expression profiles in the TCGA-LIHC database. The top 50 genes were shown in the heatmap (Fig. 5A, B). Next, genes were scored based on the correlation and then subjected to GSEA analysis. GSEA result revealed that miR-21-4-5p expression was negatively correlated with Th17 cell differentiation and the immune response-related pathways in HCC. (Fig. 5C–E).

**Fig. 6:**
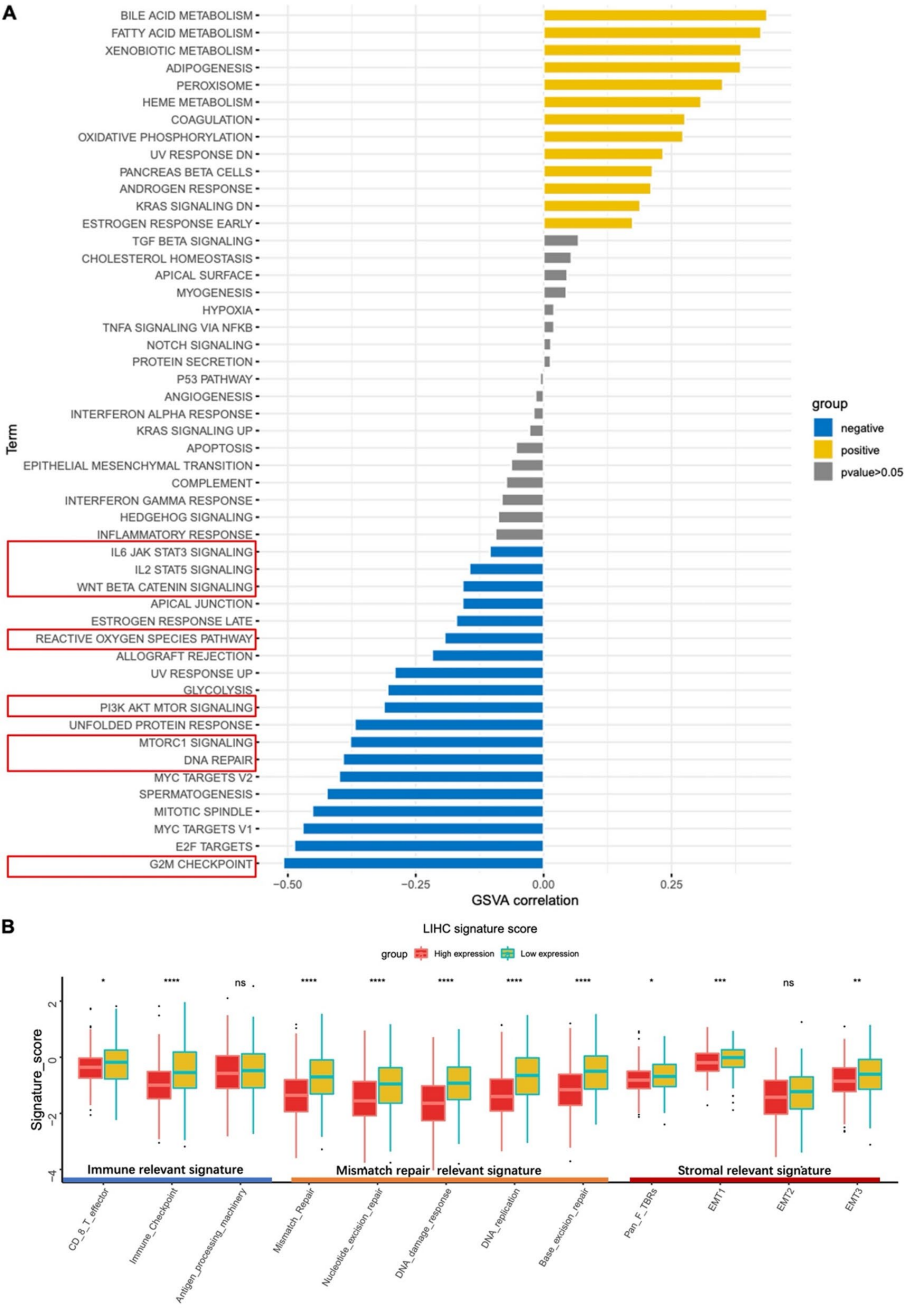
Potential pathways related to miR-24-1-5p expression in HCC. (**A**) GSVA results based on Hallmark gene sets enrichment analysis of miR-24-1-5p in tumor samples of TCGA-LIHC. (**B**) Comparison of signature scores for different tumor microenvironment gene sets between TCGA-LIHC samples with high and low miR24-1-5p expression levels. *p < 0.05; **p < 0.01; ***p < 0.001; ****p < 0.0001

To further clarify the functions of miR-24-1-5p in HCC, we conducted GSVA analysis using the Hallmark gene sets. As shown in Fig. 6A, miR-24-1-5p expression was negatively correlated with tumor-related immune suppression pathways such as the IL2/STAT5 and IL6/JAK/STAT3 signaling pathways. In addition, pathways related to tumor proliferation (WNT/Beta-Catenin, PI3K/AKT/mTOR, MTORC1, MYC targets V1) and pathway related to DNA mismatch repair (DNA repair) also showed significant negative correlations with miR-24-1-5p expression. Our result suggests that miR-24-1-5p expression in HCC is negatively correlated with tumorigenesis and cancer progression.

Based on the close association between exosomes and the tumor microenvironment, we further analyzed the correlation between miR-24-1-5p and the gene sets related to the tumor microenvironment. Of the 12 gene sets tested, tumor microenvironment-score analysis showed that miR-24-1-5p was negatively correlated with 10 gene sets (*p* < 0.05) (Fig. 6B), suggesting that miR-24-1-5p is involved in the regulation of tumor immune microenvironment.

### Correlation between the mir-24-1-5p targets and HCC prognosis

**Fig. 7:**
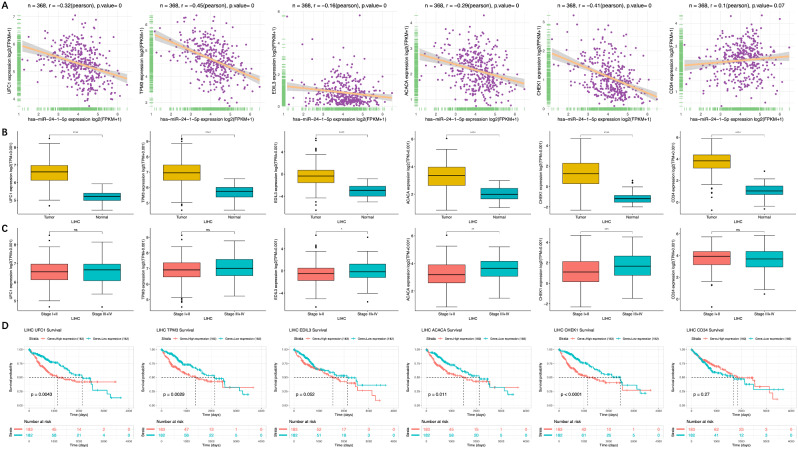
The correlation between miR-24-1-5p targets and prognosis of HCC. (**A**) Correlation analysis of miR-24-1-5p and its target genes in TCGA-LIHC cohort. (**B**) Expression patterns of the target genes between tumor and normal tissues in TCGA-LIHC cohort. (**C**) Expression of the target genes in different pathological stages in TCGA-LIHC cohort. (**D**) Correlation of the target genes with median survival in TCGA-LIHC cohort. *p < 0.05; **p < 0.01; ***p < 0.001; ****p < 0.0001

We hypothesized a negative correlation between miR-24-1-5p and its target genes based on the fact that miRNA suppresses target gene expressions. Thus, we conducted an expression correlation analysis using the TCGA-LIHC data. All target genes, except CD34, were negatively correlated with miR-24-1-5p (Fig. 7A). We noted that gene expression was regulated not only by miRNA but also by other regulatory mechanisms, such as the copy number variation (CNV) and the degree of DNA methylation. The CNV and methylation analyses on the 6 target genes in HCC using GSCA showed a higher proportion of CNV in tumor (Figure S3A). The CNV was positively correlated with the mRNA expression (Figure S3B). The methylation difference analysis between tumor and normal tissues showed that all 5 gene loci, except UFC1, were hypomethylated (Figure S3C). There was no relevant data on UFC1 methylation difference in GSCA. The methylation levels were negatively correlated with the mRNA expression (Figure S3D). These data indicated that the six target genes of miR-24-1-5p, along with CNV and DNA methylation, potentially play a complex role in the progression of HCC.

Next, we determined the expression and prognostic value of these target genes in the TCGA-LIHC database. As shown in Fig. 7B, the expressions of these 6 target genes were significantly higher in HCC compared to the normal tissue (*p* < 0.0001). The increased expressions of ACACA, CHEK1, and EDIL3 were significantly associated with advanced-stage tumors (*p* < 0.05) (Fig. 7C). Survival analysis also showed that HCC patients with high expression of ACACA, CHEK1, TPM3, or UFC1 had poorer survival compared to those with low expressions (*p* = 0.011, *p* < 0.0001, *p* = 0.029, and *p* = 0.0043 respectively) (Fig. 7D). Taken together, these results indicate that these miR-24-1-5p targets are associated with poor prognosis in HCC patients.

### Potential mechanisms by which the mir-24-1-5p targets accelerate the progression of HCC

To determine the potential mechanisms of the miR-24-1-5p targets in HCC, we performed GSVA using the hallmark gene sets. As shown in Figure S4, these 6 target genes were positively correlated with pathways related to tumor progression, such as the WNT/Beta-Catenin and PI3K/AKT/mTOR signaling pathways. Similarly, all target genes, except CD34, were positively correlated with signaling pathways related to cell proliferation, differentiation, and survival (G2/M checkpoint, Myc targets V1). In addition, CD34, EDIL3, and TPM3 were positively correlated with signaling pathways related to tumor immune response, invasion, and metastasis, particularly the IL6/JAK/STAT3, inflammatory response, TNF-α signaling via NF-kB, and epithelial-mesenchymal transition.

**Fig. 8:**
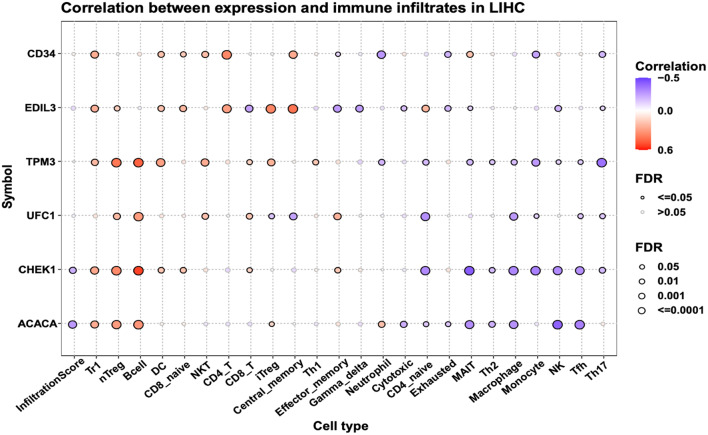
Immune cell infiltration analyses of ACACA, CHEK1, EDIL3, TPM3, UFC1, and CD34 in HCC

As our result suggests that the target genes of miR-24-1-5p are associated with immune pathways, we further analyzed the association of these target genes with immune cell infiltration using the GSCA database. The results indicated a negative correlation between ACACA, CHEK1, and the Infiltration Score, suggesting a potential suppression of the immune response in HCC. These target genes are positively correlated with the infiltration of Tr1 cells, nTreg cells, and B cells while negatively correlated with the infiltration of Th2 cells, macrophage cells, monocytes, NK cells, Tfh cells, and Th17 cells (Fig. 8). Our results suggest that the target genes of miR-24-1-5p may further drive HCC progression by recruiting pro-tumor-associated immune cells, such as Tr1 cells and nTreg cells while inhibiting the infiltration of tumor-suppressive immune cells, such as Th2 cells, NK cells, Tfh cells, and Th17 cells.

## Discussion

Identifying validated biomarkers is crucial for personalized treatment in liver surgery for HCC, especially in anesthetics selection [29]. Plasma exosomal miRNAs have gained popularity in cancer prognosis [30]. In our study, we profiled exosomal miRNAs in HCC patients receiving propofol or sevoflurane, revealing 35 distinct DEmiRNAs regulated exclusively by either propofol or sevoflurane. TCGA-LIHC database analysis linked 8 DEmiRNAs to HCC, including propofol-triggered miR-452-5p and let-7c-5p, and sevoflurane-induced miR-24-1-5p, miR-122-5p, miR-200a-3p, miR-4686, miR-214-3p, and miR-511-5p. Among these, upregulation of miR-24-1-5p consistently associated with lower histological grades, early-stage tumors, and higher survival; higher miR-452-5p was linked to HCC progression and poorer survival. GSEA and GSVA analyses suggested that miR-24-1-5p, along with its target genes, played a role in the tumor immune microenvironment. Gene cluster analysis of miR-24-1-5p targeted genes was negatively correlated with carcinogenic signaling pathways. Further bioinformatics analysis of miR-24-1-5p targeted genes confirmed their associations with tumor immune cell infiltration and microenvironment transformation.

To our knowledge, this is the first study to conduct an in-depth analysis on exosomal miRNAs affected by sevoflurane and propofol anesthesia in HCC. The 8 associated DEmiRNAs were miR-452-5p, let-7c-5p (propofol-triggered), and miR-24-1-5p, miR-122-5p, miR-200a-3p, miR-4686, miR-214-3p, and miR-511-5p (sevoflurane-induced). Consistently, downregulation of hepatocyte-specific miR-122 was linked to liver cancer metastasis. MiR-122 has a functional role in the TGFβ pathway with species-specific target sites [31]. Inhibiting miR-122-5p reduces inflammation and oxidative stress in liver cells independently of insulin resistance-mediated pathways [32]. miR-200a-3p can target the miR-181-5p/HOXB5/EGFR signaling pathway may provide new options for the treatment strategies of HCC [33]. Exosomal miR-200b-3p from hepatocytes suppresses endothelial ERG expression, promoting angiogenesis in HCC tissues [34]. MiR-214 promotes ferroptosis in hepatoma cells by inhibiting ATF4, a potential therapeutic target [35]. MiR-214-3p suppresses HCC progression by down-regulating MELK expression, a potential therapeutic target [36]. MiR-511-5p, expressed at low levels in HCC tissues, negatively correlates with HLTF, accelerating HCC cell growth and metastasis [37]. Our findings suggested the important and versatile roles of these miRNAs in HCC progression.

Analysis of the TCGA-LIHC database revealed that high expression of miR-24-1-5p in HCC tissues was linked to improved long-term outcomes. Previous study have confirmed miR-24-1-5p targets genes involved in key pathways such as the cell cycle, AMPK signaling, Hippo signaling, and insulin signaling, affecting cancer cell proliferation, metastasis and apoptosis in HCC [38]. However, the effects of miR-24-1-5p vary across different types of tumors. Recent studies have demonstrated that miR-24-1-5p has the capability to initiate a negative regulatory loop for β-catenin, presenting a potential strategy for combating colorectal cancer driven by β-catenin signaling [39]. The elevated expression of miR-24-1-5p in prostate cancer, however, may augment the risk of recurrence [40]. miR-24 can enhance the expression of oncogenes like cMyc, BCL2, and HIF1, while downregulating tumor suppressors such as p21 and p53 [41]. Moreover, overexpression of miR-24-1-5p leads to increased p-JNK/JNK and p-c-Jun/c-Jun ratios, which can be reversed through co-overexpression with ubiquitin [42]. Increased miR-24-1-5p expression was associated with the suppression of HCC, making it a promising target for cancer research and therapy. Nevertheless, the influence of exosomal miR-24-1-5p on HCC outcomes remains uncertain. Ye et al. demonstrated that exosomal miR-24-3p inhibited T-cell function in nasopharyngeal carcinoma [43]. This indicates that the increase of exosomal miR-24-1-5p induced by sevoflurane could impact the prognosis of HCC by regulating immune cell function. Further research is necessary to establish whether it can decrease HCC recurrence and distant metastasis, as well as improve long-term survival.

Gene cluster analysis of the genes influenced by miR-24-1-5p in HCC showed a negative correlation with carcinogenic signaling pathways related to tumor proliferation and immunity. Our observation aligns with previous studies that suggested sevoflurane can regulate tumor proliferation and immunological signaling pathways [44, 45]. Further bioinformatics analysis of miR-24-1-5p targets, including CD34, ACACA, TPM3, UFC1, EDIL3, and CHEK1, reinforces their role in tumor immune cell infiltration and the transformation of the tumor microenvironment. CD34 helps distinguish benign and malignant hepatocellular lesions [46], and high expression of CD34-positive cells increases HCC risk [47]. ACACA is a crucial HCC prognostic factor, impacting cell proliferation, migration, and immune cell infiltration through Wnt/β-catenin signaling [48]. TPM3 upregulation contributed to liver cancer development [49]. LincRNA-UFC1 promoted tumor growth by interacting directly with the mRNA stabilizing protein HuR to regulate levels of β-catenin in HCC cells [50]. EDIL3 is a novel epithelial-mesenchymal transition (EMT) regulator, promoting angiogenesis, metastasis, and recurrence in HCC by activating ERK and TGF-β signaling [51], and inhibiting anoikis [52]. CHEK1 overexpression in HCC correlated with poor survival [53]. Its regulation involved miR-195 and IFN-γ, affecting apoptosis and tumor NK cell infiltration [54]. In summary, miR-24-1-5p targets play diverse roles in HCC progression, affecting immune responses and the tumor microenvironment.

Target genes of miR-24-1-5p may drive HCC progression by recruiting pro-tumor-associated immune cells like Tr1 and nTreg cells, while inhibiting the infiltration of tumor-suppressive immune cells such as Th2 cells, NK cells, Tfh cells, and Th17 cells. Tr1 cells were associated with intra-tumoral immunosuppression in HCC, with pDCs promoting their activity through ICOS-L-induced IL-10 production [55]. GDF15 promoted the generation and function of Treg cells, contributing to HCC-related immunosuppression. It interacted with CD48 on T cells, downregulating STUB1 and enhancing nTreg cell suppressive function [56]. The enrichment of Th2 cells in HCC may be mediated by STAT5A signaling, facilitating tumor growth or metastasis through immunosuppressive cytokines like IL-4 and IL-10 [57]. As a subset of CD4^+^ T helper cells expressing RORγ and IL-17, Th17 cells were linked to chronic inflammation, tumorigenesis, and HCC development. They impact liver inflammation through IL-17, while IL-22 supports hepatocyte survival [58]. Zhang et al. reported elevated intratumorally Th17 cells in HCC, predicting poorer outcomes [59]. Originating from naive CD4^+^ T cells, Tfh cells secrete IL-21, IL-4, and CXCL13, essential for B cell function [60]. In HCC patients, both circulating and tumor infiltrating Tfh cells were reduced, correlating with poorer prognosis [61]. NK cells play an anti-tumor role in HCC control, and a decline in IFN-γ producing NK cells can increase HCC recurrence rates. Disruption of immune surveillance by NK cells signified the onset of HCC development, as observed in a mouse model of liver cancer [62]. In summary, miR-24-1-5p targeted genes influence HCC progression by modulating the immune cell composition such as T cell subtypes and NK cells within the tumor microenvironment, favoring pro-tumor immune cells and hampering tumor-suppressive immune cells.

A limitation of our study lies in its reliance on correlational analysis, which necessitates experimental validation to establish causal relationships between anesthetic-associated exosomal miRNAs and tumor progression. Due to the limited accessibility of publicly available datasets concerning exosome-associated miRNA and the role of exosomes in tumor prognosis, particularly their influence on the immune microenvironment of tumor cells, we opted to conduct an exploratory analysis of exosome-specific miRNA using the TCGA database, which provides a substantial sample size and clinical follow-up data. Additionally, the small sample size prevented the validation of identified miRNAs and related signaling pathways via molecular biology experiments. Furthermore, our findings may have limited generalizability as only male patients with early-stage HCC were included as participants. To enhance the robustness of our conclusions, future investigations should encompass a more extensive HCC patient cohort.

## Conclusions

Our study demonstrates that exosomal miRNA signatures in HCC patients change depending on the anesthetic procedure during laparoscopic hepatectomy. The bioinformatic analysis suggests that miR-24-1-5p, which is upregulated by sevoflurane in plasma exosomes, may have a potential impact on the immune microenvironment of HCC and be related to better outcomes. Our findings may provide valuable insights into the role of circulating exosomes influenced by anesthetics during tumor surgery.

### Electronic supplementary material

Below is the link to the electronic supplementary material.

Supplementary Material 1

## Data Availability

The TCGA-LIHC dataset is available in the Sangerbox repository (http://sangerbox.com/)[63]. The Hallmark gene sets of MsigDB database is available in https://www.gsea-msigdb.org/gsea/msigdb. The GSCA is available in http://bioinfo.life.hust.edu.cn/GSCA/[27]. The high-throughput data produced in this study has been effectively deposited in the Sequence Read Archive (SRA) of the National Center for Biotechnology Information (NCBI) under the Bioproject accession PRJNA1078077 (https://www.ncbi.nlm.nih.gov/bioproject/PRJNA1078077).

## Abbreviations

HCC: Hepatocellular Carcinoma
miRNA: MicroRNA
DEmiRNAs: Differentially expressed microRNAs
TCGA-LIHC: The Cancer Genome Atlas-Liver Hepatocellular Carcinoma
GO: Gene Ontology
KEGG: Kyoto Encyclopedia Of Genes And Genomes
GSEA: Gene Set Enrichment Analysis
GSVA: Gene Set Variation Analysis
GSCA: Gene Set Cancer Analysis
CNV: Copy Number Variation
