# Supplementary material for: Uncovering essential anesthetics-induced exosomal miRNAs related to hepatocellular carcinoma progression: a bioinformatic investigation
Source: BMC Med Genomics. 2024 Jun 5;17:154. doi: 10.1186/s12920-024-01922-7 (PMC11155038; doi:10.1186/s12920-024-01922-7)
Supplement: Supplementary file 1 — Supplementary Material 1 [file 12920_2024_1922_MOESM1_ESM.docx]

**Supplemental Table and Figures**

**Table S1.** **DEmiRNAs significantly regulated by propofol and sevoflurane.** baseMean: mean miRNA expression; log_2_FC: log_2_ fold change; *DEmiRNA exclusively regulated by either anesthetic agent.

| **Regulation** | **miRNA** | **baseMean** | **Log_2_FC** | ***p*-value** |
| --- | --- | --- | --- | --- |
| Propofol-up | hsa-miR-1246 | 17241.14 | 4.48 | 1.79E-07 |
|  | hsa-miR-1290 | 2819.48 | 3.90 | 9.51E-06 |
|  | hsa-miR-452-5p* | 167.22 | 3.21 | 1.16E-03 |
|  | hsa-miR-125b-2-3p | 285.53 | 2.80 | 1.86E-03 |
|  | hsa-miR-206* | 61.14 | 2.66 | 9.78E-04 |
|  | hsa-miR-193a-5p | 2855.56 | 2.50 | 3.86E-03 |
|  | hsa-miR-885-3p | 57.08 | 2.45 | 4.47E-03 |
|  | hsa-miR-214-5p | 53.97 | 2.33 | 1.51E-03 |
|  | hsa-miR-99a-5p | 127551.23 | 2.20 | 7.34E-05 |
|  | hsa-miR-148a-3p | 843070.84 | 2.08 | 5.71E-04 |
|  | hsa-miR-203a-3p | 61.73 | 2.08 | 7.91E-03 |
|  | hsa-miR-378d* | 1392.72 | 2.07 | 5.09E-03 |
|  | hsa-miR-542-3p | 50.25 | 1.91 | 8.52E-03 |
|  | hsa-miR-30a-5p | 4448.07 | 1.68 | 7.58E-03 |
| Propofol-down | hsa-miR-1307-3p | 7779.10 | -1.77 | 6.98E-03 |
|  | hsa-let-7a-5p* | 70598.34 | -1.80 | 2.74E-03 |
|  | hsa-miR-92b-3p* | 218.16 | -2.03 | 4.67E-03 |
|  | hsa-let-7f-5p* | 42403.02 | -2.10 | 3.12E-03 |
|  | hsa-let-7c-5p* | 6514.70 | -2.22 | 2.02E-03 |
|  | hsa-miR-92a-3p* | 91166.35 | -2.32 | 1.25E-04 |
|  | hsa-let-7b-5p | 127730.88 | -2.69 | 1.00E-05 |
|  | hsa-miR-4433b-3p* | 16569.40 | -5.91 | 8.66E-08 |
| Sevoflurane-up | hsa-miR-1246 | 30384.28 | 5.52 | 1.02E-22 |
|  | hsa-miR-1290 | 5653.77 | 5.42 | 1.47E-14 |
|  | hsa-miR-4686* | 86.54 | 4.91 | 6.35E-12 |
|  | hsa-miR-214-3p* | 82.75 | 3.27 | 8.47E-06 |
|  | hsa-miR-214-5p | 102.48 | 3.13 | 1.65E-05 |
|  | hsa-miR-125b-2-3p | 456.94 | 2.76 | 1.41E-04 |
|  | hsa-miR-885-3p | 95.92 | 2.75 | 2.61E-04 |
|  | hsa-miR-95-3p* | 215.86 | 2.74 | 2.12E-04 |
|  | hsa-miR-194-5p* | 17148.35 | 2.73 | 4.93E-04 |
|  | hsa-miR-499a-5p* | 241.58 | 2.65 | 6.94E-04 |
|  | hsa-miR-99a-5p | 230779.70 | 2.51 | 6.56E-09 |
|  | hsa-miR-455-5p* | 732.82 | 2.51 | 1.08E-03 |
|  | hsa-miR-148a-3p | 1784801.23 | 2.31 | 5.14E-06 |
|  | hsa-miR-218-5p* | 92.74 | 2.31 | 9.70E-04 |
|  | hsa-miR-203a-3p | 89.59 | 2.30 | 2.40E-03 |
|  | hsa-miR-548ah-3p* | 245.62 | 2.22 | 2.98E-03 |
|  | hsa-miR-548p* | 122.72 | 2.22 | 2.41E-03 |
|  | hsa-miR-24-1-5p* | 95.18 | 2.15 | 4.08E-03 |
|  | hsa-miR-27b-3p* | 19931.81 | 2.13 | 4.53E-05 |
|  | hsa-miR-30a-5p | 7953.68 | 2.06 | 8.45E-08 |
|  | hsa-miR-100-5p* | 15270.15 | 1.97 | 8.29E-07 |
|  | hsa-miR-542-3p | 73.97 | 1.91 | 8.42E-03 |
|  | hsa-miR-511-5p* | 606.50 | 1.89 | 1.91E-04 |
|  | hsa-miR-122-5p* | 2865990.07 | 1.88 | 1.54E-04 |
|  | hsa-miR-193b-5p* | 1148.35 | 1.73 | 1.01E-03 |
|  | hsa-miR-215-5p* | 2285.83 | 1.72 | 2.18E-04 |
|  | hsa-miR-192-5p* | 79351.72 | 1.63 | 3.00E-03 |
|  | hsa-miR-152-3p* | 1774.22 | 1.50 | 7.85E-04 |
|  | hsa-miR-200a-3p* | 324.94 | 1.32 | 3.56E-03 |
|  | hsa-miR-193a-5p | 3479.47 | 1.26 | 5.46E-03 |
| Sevoflurane-down | hsa-miR-1307-3p | 9321.46 | -1.25 | 7.79E-04 |
|  | hsa-miR-16-2-3p* | 8251.14 | -1.35 | 2.77E-03 |
|  | hsa-miR-370-3p* | 1479.83 | -1.62 | 2.53E-03 |
|  | hsa-miR-4446-3p* | 434.77 | -1.73 | 6.16E-03 |
|  | hsa-miR-760* | 412.64 | -1.78 | 2.52E-03 |
|  | hsa-let-7b-5p | 103670.05 | -1.96 | 8.51E-04 |
|  | hsa-miR-5189-5p* | 83.04 | -2.11 | 7.26E-03 |
|  | hsa-miR-4429* | 240.96 | -2.41 | 4.34E-03 |
|  | hsa-miR-4489* | 51.22 | -2.85 | 1.30E-03 |


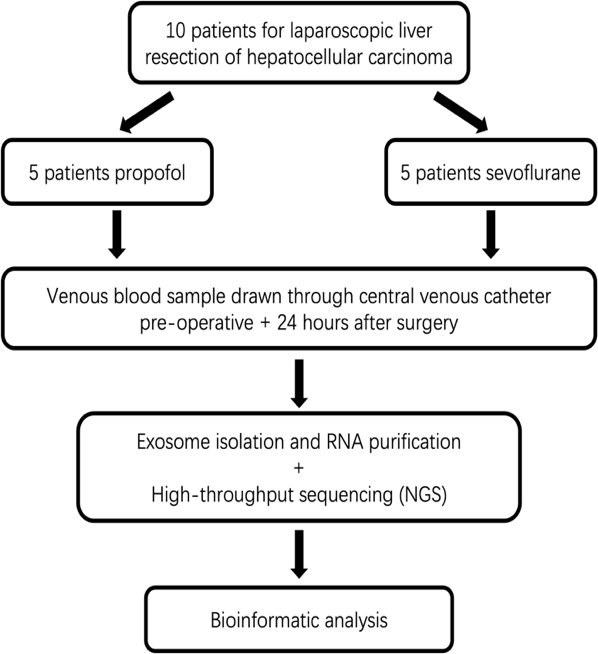


**Figure S1. Description of the patient recruitment and selection process.** In both groups, exosomes were isolated from pre- and post-operative serum. The exosomal miRNA profile was then assessed using next-generation sequencing (NGS). After normalization, the clean reads were used for subsequent bioinformatic analysis.


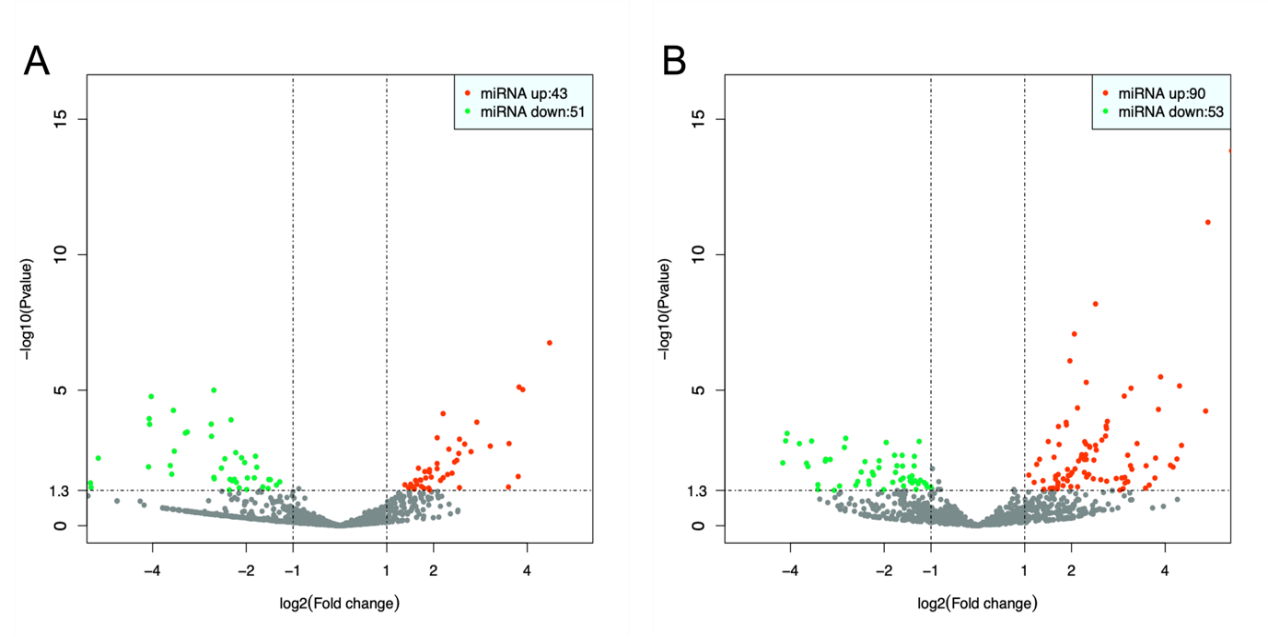
 **Figure S2. Volcano map of DEmiRNAs.** (A) Volcano map of propofol group post-operative vs. pre-operative. (B) Volcano map of sevoflurane group post-operative vs. pre-operative.


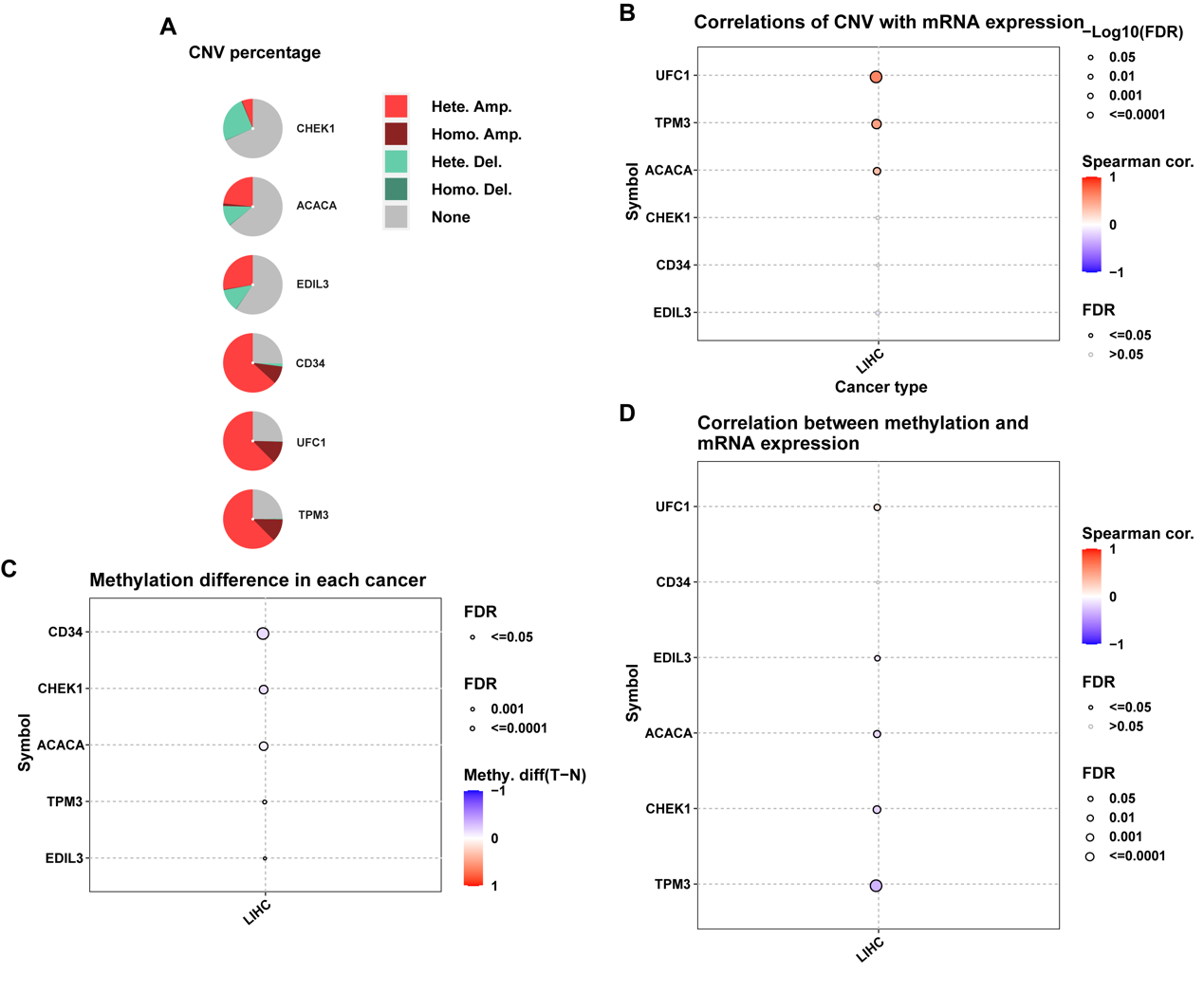


**Figure S3. Mutation analysis of the miR-24-1-5p targets using GSCA.** (A) Pie plot summarizes the CNV of target genes in HCC. (B) Correlations between CNV and mRNA expression of target genes in HCC. (C) Methylation difference between tumor and normal samples of target genes (except UFC1, no relevant data on methylation difference in GSCA) in HCC. (D) Correlations between methylation and mRNA expression of target genes in HCC.


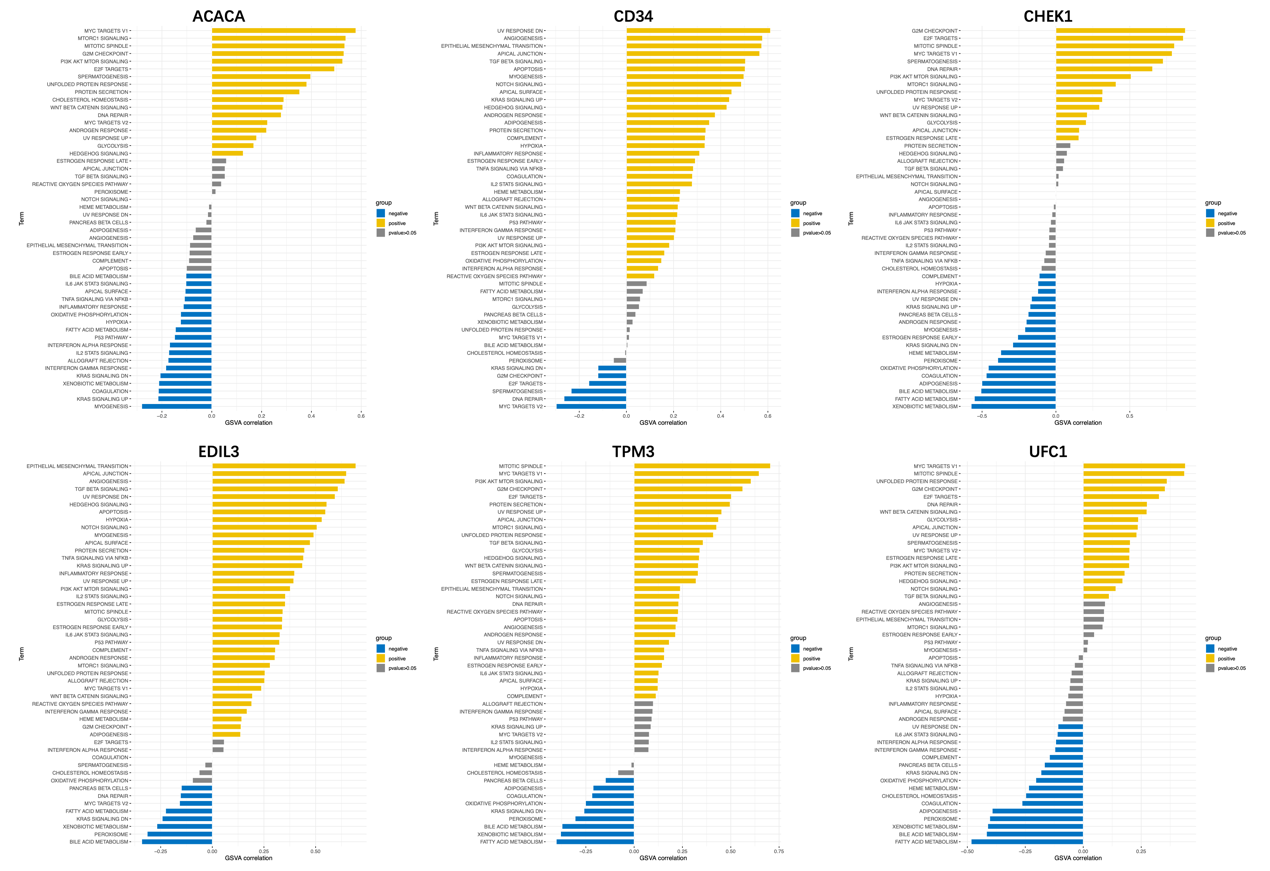


**Figure S4. GSVA results based on Hallmark gene sets enrichment analysis of the miR-24-1-5p targets in tumor samples of TCGA-LIHC.**
